# Supplementary material for: Transcriptomic Analyses of the Hypothalamic-Pituitary-Gonadal Axis Identify Candidate Genes Related to Egg Production in Xinjiang Yili Geese
Source: Animals (Basel). 2020 Jan 6;10(1):90. doi: 10.3390/ani10010090 (PMC7023467; doi:10.3390/ani10010090)
Supplement: Supplementary file 1 [file animals-10-00090-s001.pdf]

Table S1 RT-PCR Primers of the differentially expressed genes

Gene	Primer Sequence	Product Length	Annealing Temperature	GenBank accession number
LOC106040733	F:AAGGAAGTAAAATGGCTCGT<br>R:GTTCCCATCTGAATCCACGTA	101bp	58℃	XM_013188822.1
NR4A3	F:TATAATCTTCTGACTGCCTCC<br>R:GCAGAATACAAACTTATCCTCA	177bp	60℃	XM_013181116.1
GPR55	F:CTCTGGGTATGGTCTATAAGC<br>R:GCTCTTTTCCAGTCTTCCGAA	179bp	60℃	XM_013188057.1
SYNPO2	F:ACCTATGCTGAAGTTATCGTT<br>R:CCATTTTCTCTTTCAGCACTCA	113bp	58℃	XM_013185299.1
MRAP	F:CCAACAGAACCAACTCGTCT<br>R:CAGAGCGGGACATATACAGCA	186bp	58℃	XM_013192843.1
SLC4A4	F:TTCTGACTGCCCCTACACA<br>R:AAGGAATTTTATCAGCACGAC	153bp	58℃	XM_013193772.1
DRD5	F:ACCACCTTTAATATCTTCGTCT<br>R:TGCTTATATTAACGGTCTCCAC	157bp	58℃	XM_013193772.1
NEU4	F:CCCTTGCCAGCCTCGGTTTAC<br>R:TGGTCTCCTTCTCGAAGAGCACAGTC	194bp	58℃	XM_013186040.1
MYL9	F:GCCCATCAACTTCACCATGTTCC<br>R:AGCTCACGCAGATGGTCCT	141bp	60℃	XM_013195939.1
IGF1	F:GATGTACTGTGCTCCAAT<br>R:TTCATTCTTGTGGATGGC	169bp	58℃	XM_013181061.1
DRD1	F:ACGGCCTTGCATTCCAGA<br>R:TGTTCTCTACTTTGGTCCCCT	197bp	58℃	XM_013191902.1
PRKCA	F:GAGACATCAGGGAACATGCTT<br>R:CCTCGTGTGAAGAACTTATCGAA	133bp	60℃	XM_013178940.1
P2RX1	F:ACTACAAAGAAGATGGGACAGAC<br>R:AATACCAATTCCAGAGCCGAT	133bp	58℃	XM_013190021.1
β-actin	F:GCTGTCTTCCCATCCATCGTG<br>R:TCTTCTCCATGTCATCCCAGT	169bp	58℃	M26111.1
SAMD3	F:AAAATGAGCCAGAACCCCTT<br>R:AAATACAGTTTCCGCTCCACA	124bp	58℃	XM_013175365.1
GAL3ST1	F:GCTCTACTCCTACGCTGTGCCTC<br>R:CCGGCAGCCTCCTCCCGAA	124bp	58℃	XM_013201212.1

Table S2. RNA Quality Determination.

sample	concentration（ng/μL）	Total amount（μg）	OD260nm/OD280nm	OD260nm/OD230nm	RIN	28s/18s
X01	435	18.27	1.877	1.914	9.1	1.1
X02	410	17.22	1.934	1.723	9.2	0.9
X03	875	36.75	1.978	2.303	9	0.9
X04	460	19.32	1.960	2.159	8.3	0.7
X21	56	5.152	1.962	1.072	8.5	1.0
X22	36	3.312	1.825	0.83	7.8	0.8
X23	350	14.7	1.978	2.142	9.1	0.9
X24	340	14.28	1.957	2.251	8.8	0.7
C01	172	7.224	1.9	1.484	9.6	0.9
C02	338	14.196	1.916	2.116	9.1	0.8
C03	194	8.148	2.035	2.275	8.9	0.9
C04	184	7.728	1.951	2.333	8.7	0.7
C21	92	3.864	1.879	1.265	8.8	0.9
C22	196	8.232	1.885	1.885	9	0.9
C23	118	4.956	2.2	1.791	8.9	0.8
C24	147	6.174	1.978	1.935	8.8	0.8
L01	1418	59.556	1.997	2.272	8.6	1.0
L02	652	27.384	2.063	2.063	8.7	1.0
L03	1222	51.324	1.99	2.01	8.6	1.3
L04	328	10.496	2.158	1.843	7.9	0.7
L21	664	27.888	1.976	2.062	8.3	1.0
L22	886	37.212	2.161	2.161	8.6	1.4
L23	1502	63.084	2.035	2.152	9	1.4
L24	585	24.570	1.918	2.18	8.3	1.1

Table S3. Sequencing Data Quality

Sample name	Raw reads	Clean reads	Clean bases	Error rate(%)	Q20(%)	Q30(%)	GC content(%)
X01	65185202	63820988	9.57G	0.02	95.16	88.61	49.92
X02	50865426	48994010	7.35G	0.02	95.44	89.16	49.13
X03	48689924	47486502	7.12G	0.02	95.64	89.53	48.30
X04	53679466	52544012	7.88G	0.02	95.89	90.01	49.19
C01	52570862	50734276	7.61G	0.02	95.31	88.98	49.11
C02	48098944	46487768	6.97G	0.02	95.43	89.18	48.84
C03	51670028	49927304	7.49G	0.02	95.10	88.57	48.75
C04	49034166	47471474	7.12G	0.02	95.52	89.31	48.28
L01	48076190	46481224	6.97G	0.02	96.18	90.58	50.39
L02	49122812	47674070	7.15G	0.02	96.54	91.26	49.45
L03	53642642	52000348	7.8G	0.02	96.20	90.64	49.49
L04	44688338	43311034	6.5G	0.02	95.57	89.45	50.57
X21	48545716	47405610	7.11G	0.02	95.32	88.89	48.45
X22	60081486	58321012	8.75G	0.02	95.51	89.40	49.57
X23	50854898	49243474	7.39G	0.02	95.88	90.00	48.33
X24	54658024	52884948	7.93G	0.02	95.81	89.90	48.76
C21	46894856	45438188	6.82G	0.02	96.13	90.51	48.30
C22	48916538	47301552	7.1G	0.02	95.46	89.24	48.58
C23	45350410	43885970	6.58G	0.02	95.95	90.17	48.57
C24	49964224	48334796	7.25G	0.02	96.04	90.39	48.69
L21	56185752	54455308	8.17G	0.02	95.52	89.34	50.17
L22	49119512	47610514	7.14G	0.02	96.21	90.72	49.98
L23	46126778	44825988	6.72G	0.02	95.29	88.60	49.87
L24	41075344	39855776	5.98G	0.02	95.48	89.21	49.82

Note:(1) raw reads: count the original sequence data, and count the sequence number of each file in four lines. (2) clean reads: the calculation method is the same as raw reads, except that the statistical file is the filtered sequencing data. The follow-up analysis of biological information is based on clean reads. (3) clean bases: the number of clean reads multiplied by the length, and converted to g. (4) error rate: calculated by Formula 1. (5) Q20 and q30: calculate the percentage of bases with the pH value greater than 20 and 30 in the total base respectively. (6) GC content: calculate the percentage of the total number of bases g and C in the total number of bases.3.2 Statistics of Sequencing Read Alignment
